# Functional KCa1.1 channels are crucial for regulating the proliferation, migration and differentiation of human primary skeletal myoblasts

**DOI:** 10.1038/cddis.2016.324

**Published:** 2016-10-20

**Authors:** Rajeev B Tajhya, Xueyou Hu, Mark R Tanner, Redwan Huq, Natee Kongchan, Joel R Neilson, George G Rodney, Frank T Horrigan, Lubov T Timchenko, Christine Beeton

**Affiliations:** 1 Department of Molecular Physiology and Biophysics, Baylor College of Medicine, Houston, TX 77030, USA; 2 Graduate Program in Molecular Physiology and Biophysics, Baylor College of Medicine, Houston, TX 77030, USA; 3 Cardiovascular Research Institute, Baylor College of Medicine, Houston, TX 77030, USA; 4 Interdepartmental Graduate Program in Translational Biology and Molecular Medicine, Baylor College of Medicine, Houston, TX 77030, USA; 5 Center for Drug Discovery, Baylor College of Medicine, Houston, TX 77030, USA; 6 Department of Pediatrics Neurology, Cincinnati Children's Hospital, Cincinnati, OH 45219, USA; 7 Biology of Inflammation Center, Baylor College of Medicine, Houston, TX 77030, USA

## Abstract

Myoblasts are mononucleated precursors of myofibers; they persist in mature skeletal muscles for growth and regeneration post injury. During myotonic dystrophy type 1 (DM1), a complex autosomal-dominant neuromuscular disease, the differentiation of skeletal myoblasts into functional myotubes is impaired, resulting in muscle wasting and weakness. The mechanisms leading to this altered differentiation are not fully understood. Here, we demonstrate that the calcium- and voltage-dependent potassium channel, KCa1.1 (BK, Slo1, *KCNMA1*), regulates myoblast proliferation, migration, and fusion. We also show a loss of plasma membrane expression of the pore-forming *α* subunit of KCa1.1 in DM1 myoblasts. Inhibiting the function of KCa1.1 in healthy myoblasts induced an increase in cytosolic calcium levels and altered nuclear factor kappa B (NF*κ*B) levels without affecting cell survival. In these normal cells, KCa1.1 block resulted in enhanced proliferation and decreased matrix metalloproteinase secretion, migration, and myotube fusion, phenotypes all observed in DM1 myoblasts and associated with disease pathogenesis. In contrast, introducing functional KCa1.1 *α*-subunits into DM1 myoblasts normalized their proliferation and rescued expression of the late myogenic marker Mef2. Our results identify KCa1.1 channels as crucial regulators of skeletal myogenesis and suggest these channels as novel therapeutic targets in DM1.

Myotonic dystrophy type 1 (DM1) is characterized by multi-systemic disorders including muscle weakness and wasting.^1, 2, 3^ In DM1, the expansion of tri-nucleotide CUG repeats results in the formation of stable hairpin structures and sequestration of splicing regulators, such as muscleblind-like proteins and CUG-binding proteins, required for pre-mRNA alternative splicing. Misregulated alternative splicing impairs function of signaling molecules, causing multi-systemic errors. Among the many disorders in DM1, muscle wasting is one of the most conspicuous.

Skeletal muscles require constant regeneration and repair through myogenesis. Skeletal myoblasts proliferate, express myogenic markers such as myoD and myf5, withdraw from the cell cycle, and upregulate differentiation proteins such as myogenin and myocyte enhancer factor (Mef2) while fusing into multinucleated myotubes.^4^ In patients with DM1, the myoblasts fail to exit the cell cycle, leading to a delay in myotube fusion.^5, 6, 7^

Ion channel loss-of-function in DM1 has been reported in regards to myotonia. Mutations in a chloride channel (*CLCN1*) and in apamin-sensitive potassium channels are linked to myotonia in DM1 patients, as is the misregulated splicing of the calcium channel Cav1.1.^8, 9, 10^ Splicing errors in Cav1.1 channels are associated with increased central nucleation and muscle weakness in DM1 patients and mouse models. However, during early steps of myogenesis, myoblasts largely express T-type calcium channels instead of Cav1.1 and provide hyperpolarization to fusion-competent myoblasts.^11^ In addition, the resting membrane potential is unchanged between normal and DM1 myoblasts.^12^ Although these electrophysiological observations in DM1 hint towards a role in myopathy, they are not sufficient to explain muscle wasting.

Potassium channels are important regulators of proliferation in many cell types.^13, 14, 15^ To understand the molecular events that lead to delayed myotube fusion, we assessed the role of potassium channels in regulating proliferation, matrix metalloproteinase (MMP) levels, migration, and fusion of skeletal myoblasts from patients with DM1 and from healthy volunteers. We show that the voltage- and calcium-activated potassium channel, KCa1.1 is a crucial regulator of myoblasts proliferation and fusion. These findings suggest a novel approach and target to treat delayed myogenesis in patients with DM1 by modulating KCa1.1.

## Results

### DM1 myoblasts express fewer functional KCa1.1 than healthy myoblasts

**Figure 1:**
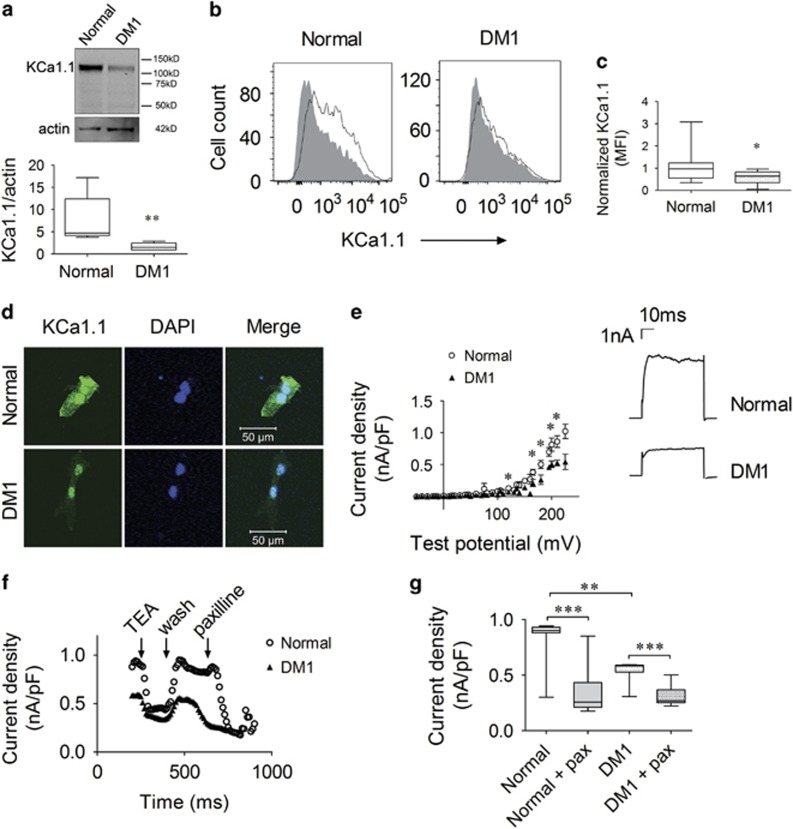
Reduced KCa1.1 channel expression and activity at the plasma membrane of DM1 myoblasts. (**a**) Top, representative western blot against the KCa1.1 *α*-subunit (~115 kD) and actin (~42 kD) on total protein extracts from normal and DM1 myoblasts. Bottom, median, and interquartile ranges of KCa1.1 *α*-protein levels normalized to actin protein levels; *n*=5 independent replicates in three normal donors and *n*=6 independent replicates in three DM1 donors; **P<0.01 by Mann–Whitney *U*-test comparing normal with DM1. (**b**) Representative flow cytometric profiles of myoblasts stained for KCa1.1 *α* (black line) and negative control (secondary antibody alone; shaded). (**c**) Median fluorescence intensities (MFI) and interquartile ranges of KCa1.1 *α* staining in normal (*n*=21 independent replicates, four donors) and DM1 (*n*=11 independent replicates, three donors) myoblasts compared with negative control; *P<0.05 by Mann–Whitney *U*-test. (**d**) Representative images of normal (top) and DM1 (bottom) myoblasts stained with antibodies against KCa1.1 *α* (green) and counterstained with DAPI (blue) to detect nuclei. Scale bar=50 *μ*m. (**e**) Current density (nA/pF) *versus* test potential (mV) plot of normal (black; *n*=13 cells) and DM1 (red; *n*=10 cells) myoblasts expressed as median and interquartile ranges. The inset shows representative whole-cell currents of normal (black) and DM1 (red) myoblasts at 200 mV. (**f**) Current density (nA/pF) of normal (black) and DM1 (red) myoblasts at 200 mV with perfusion of TEA (1 mM), Na-Ringer wash, and paxilline (200 nM). (**g**) Quantification of the effects of paxilline on the current density of DM1 (black) and normal (white) myoblasts (*n*=8 independent replicates, three donors each) expressed as median and interquartile ranges. **P<0.01; ***P<0.001 by Kruskal–Wallis test with Dunn's multiple comparisons

We first assessed the expression of pore-forming *α*-subunits of KCa1.1 in skeletal myoblasts isolated from healthy subjects and from patients with DM1. Protein levels of KCa1.1 *α*, assessed by immunoblotting, were lower in DM1 myoblasts than in normal cells (Figure 1a). Flow cytometry (FCM) confirmed that normal myoblasts express significantly higher levels of KCa1.1 *α* than do DM1 myoblasts (Figures 1b and c). We next used immunostaining to visualize the subcellular localization of KCa1.1 *α*-subunits in myoblasts. Normal myoblasts displayed KCa1.1 *α* both at the plasma membrane and in the nucleus, whereas DM1 myoblasts only expressed it in the nucleus (Figure 1d,Supplementary Video 1 and Video 2).

Whole-cell patch-clamp analysis of myoblasts showed that the current density at the plasma membrane of normal myoblasts is higher than that of DM1 myoblasts above 160 mV (Figure 1e), a membrane potential range at which KCa1.1 opens in the absence of intracellular calcium.^16^ We next tested the effects of the nonselective potassium channel blocker tetraethylammonium (TEA) and of the selective KCa1.1 blocker paxilline^17^ on the currents elicited at 200 mV. Both blockers inhibited the potassium current and significantly reduced peak current density (Figure 1f). Interestingly, paxilline reduced the potassium current density by 85% in normal myoblasts but only by 23% in DM1 myoblasts (Figure 1g). This confirms the higher expression of KCa1.1 by normal myoblasts compared with DM1 myoblasts and suggests the expression of other potassium channels in the latter.

Taken together, these results show that normal myoblasts express functional KCa1.1 at their plasma membrane and that this expression is reduced in DM1 myoblasts. The analysis of mRNA levels of KCa1.1 *α* showed no significant differences between normal and DM1 myoblasts and remained stable with actinomycin D treatment (Supplementary Figure S1), suggesting that the observed difference in protein expression are not the result of differences in mRNA expression or stability.

### Loss of KCa1.1 function by normal myoblasts alters intracellular calcium levels, leading to NF*κ*B activation

**Figure 2:**
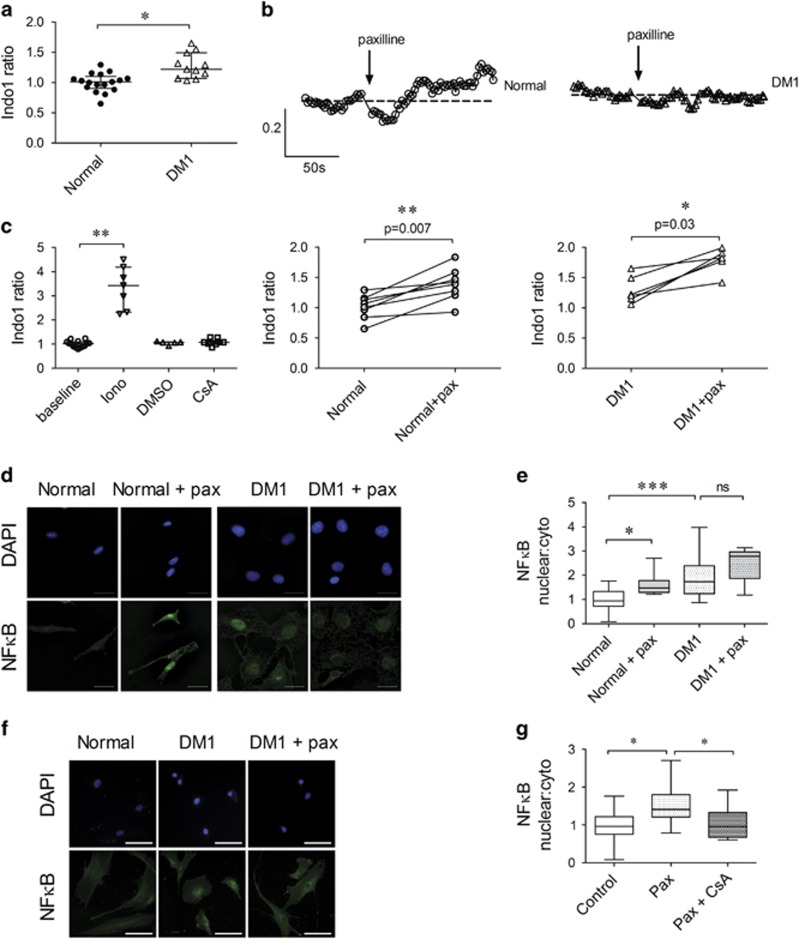
Loss of KCa1.1 function alters intracellular calcium levels, leading to NF*κ*B activation. (**a**) Basal intracellular calcium levels in normal and DM1 myoblasts detected with the ratiometric dye Indo-1-AM expressed as mean±S.E.M. *N*=8 independent replicates, three donors *P<0.05 by Mann–Whitney *U*-test. (**b**) Top: representative traces of ratiometric Indo-1 measurements in normal (left) and DM1 (right) myoblasts treated with paxilline (10 *μ*M) at indicated times. Bottom: the maximum calcium influx elicited by paxilline addition compared with average baseline Indo-1 ratio in normal (left) and DM1 (right) myoblasts; *n*=6 independent experiments. *P<0.05; **P<0.01 by Wilcoxon matched-pairs test. (**c**) Basal intracellular calcium signals were collected for 2 min and represented as baseline. Normal myoblasts treated with ionomycin (iono, 1 *μ*M; *n*=7 replicates), DMSO (*n*=5 replicates), cyclosporin A (CsA, 2.5 *μ*M; *n*=10 replicates) were measured for Indo-1 signal expressed as median and interquartile ranges. **P<0.01 by Mann–Whitney *U*-test comparing calcium influx of treated group to the baseline. (**d**) Representative images of myoblasts incubated for 16 h with paxilline (10 *μ*M) or its vehicle and stained with antibodies against NF*κ*B (green) and with DAPI (blue). Scale bar=25 *μ*m. (**e**) Median and interquartile ranges of the nuclear: cytoplasmic ratio of NF*κ*B in normal (black; *n*=8 independent replicates, four donors) and DM1 (white; *n*=5 independent replicates, three donors) myoblasts (five images were analyzed per slide per donor). *P<0.05; ***P<0.001; NS, not significant by Kruskal–Wallis test with Dunn's multiple comparisons. (**f**) Representative images of normal myoblasts: control, treated with paxilline (pax; 10 *μ*M), or treated with pax (10 *μ*M) and CsA (2.5 *μ*M), and stained with antibody against NF*κ*B (green) and DAPI (blue) to visualize nuclei. Scale bar=100 *μ*m. (**g**) Median and interquartile ranges of the nuclear: cytoplasmic ratio of NF*κ*B in normal myobalsts (*n*=3 donors; three to six images per donor). *P<0.05; **P<0.01 by Kruskal–Wallis test with Dunn's multiple comparisons

Potassium channels regulate calcium homeostasis in various cells.^18, 19^ In addition, elevated intracellular calcium levels cause myofiber degeneration in skeletal muscle.^10, 20^ We compared intracellular calcium levels in normal and DM1 myoblasts using a ratiometric calcium dye, Indo1. To record the basal intracellular calcium levels and the effects of paxilline in large cell population for a prolonged duration, we recorded continuously by FCM.^21, 22^ DM1 myoblasts displayed a significantly higher Indo-1 ratio than normal myoblasts (Figure 2a). Blocking KCa1.1 with paxilline induced an increase in intracellular calcium levels in normal myoblasts and to a lower extent in DM1 myoblasts (Figure 2b). Ionomycin was used as a positive control to elicit maximum calcium level; DMSO was a negative control (Figure 2c). The paxilline-mediated calcium influx measured by FCM was further confirmed by microscopy in some samples (Supplementary Figures S2a, b). To avoid calcium capacitative entry,^23^ assays were performed in the presence of EGTA. The addition of EGTA prevented the paxilline-induced increase in intracellular calcium levels (Supplementary Figure S2c).

As elevated levels of intracellular calcium can lead to apoptosis, we verified that blocking KCa1.1 in myoblasts with paxilline used at doses equivalent to or higher than those used in our functional assays did not induce cell death (Supplementary Figure S2d).

The activity of the transcription factor nuclear factor kappa B (NF*κ*B) is elevated in muscle wasting diseases.^24, 25, 26^ NF*κ*B is also a well-characterized regulator of myoblast proliferation and differentiation.^27^ In addition, NF*κ*B activation is led by constitutively activated calcineurin in C2C12 myoblasts.^28^ As DM1 myoblasts exhibit a higher calcium level than normal myoblasts and KCa1.1 block induced an increase in intracellular calcium levels in normal myoblasts, we compared the levels of activated NF*κ*B in normal and DM1 myoblasts; DM1 myoblasts expressed higher levels of active NF*κ*B, as shown through higher nuclear to cytoplasmic ratios (Figures 2d and e). Furthermore, paxilline-treated normal myoblasts exhibited an increase in activated NF*κ*B with no change in DM1 myoblasts treated with paxilline (Figures 2d and e). To assess whether the KCa1.1 blocker-induced upregulation of activated NF*κ*B in normal myoblasts is mediated by calcium-activated calcineurin, we tested the effects of the calcineurin inhibitor, cyclosporin A (CsA), on this upregulation. The paxilline-induced increase in activated NF*κ*B levels was prevented by CsA in normal myoblasts (Figures 2f and g). Although CsA can increase intracellular calcium levels in coronary myocytes,^29^ it did not affect these levels in skeletal myoblasts in the absence of paxilline (Figure 2c).

### Reduction of MMP-2 levels in the culture supernatants after KCa1.1 block in normal myoblasts

**Figure 3:**
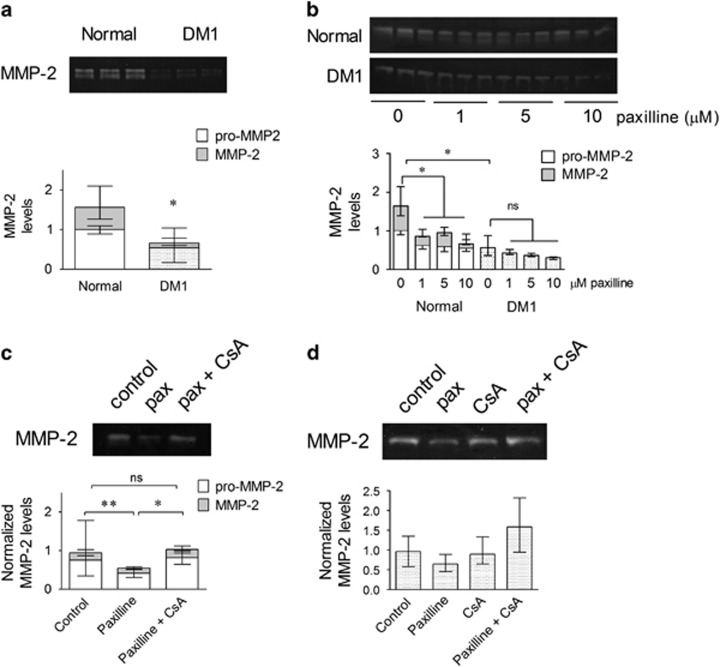
Reduction in MMP-2 culture supernatant levels after KCa1.1 block in normal myoblasts. (**a**) Top, representative gelatin zymogram of pro-MMP-2 (~70 kD; white) and active MMP-2 (~65 kD; gray) in normal and DM1 myoblast culture supernatants. Bottom, median and interquartile ranges of total MMP-2 in normal and DM1 myoblast culture supernatants (*n*=6 independent replicates, three donors per group). *P<0.05 by Mann–Whitney *U*-test. (**b**) Representative gelatin zymogram of myoblast culture supernatants treated with paxilline and quantification expressed as median and interquartile ranges (*n*=3 independent replicates, three donors per group). *P<0.05; NS, not significant by Kruskal–Wallis test with Dunn's multiple comparisons. (**c**) Representative gelatin zymogram of normal myoblast culture supernatants treated with paxilline (pax; 10 *μ*M), or with both paxilline (10 *μ*M) and CsA (2.5 *μ*M) and their quantification as median and interquartile ranges (*n*=5 independent replicates, three donors). *P<0.05; **P<0.01; NS, not significant by Kruskal–Wallis test with Dunn's multiple comparisons. (**d**) Representative gelatin zymogram of DM1 myoblast culture supernatants treated with paxilline (pax; 10 *μ*M), or CsA (2.5 *μ*M) or with both paxilline and CsA and their quantification as median and interquartile ranges (*n*=4 independent replicates, two donors)

MMPs have a key role in the migration and differentiation of many cells, including skeletal myoblast fusion into myotubes.^30, 31^ An increase in MMP-2 during migration parallels an increase in the early myotube fusion marker myogenin.^32^ In addition, MMP-2 activity is increased during myoblast fusion and the loss of MMP-2 expression leads to a delay in myoblast fusion.^30, 31^ Finally, a large calcium influx leads to reduced MMP-2 production in cancer cells.^33^ We therefore determined whether the KCa1.1-induced alteration in calcium homeostasis affects MMP-2 levels in myoblast culture supernatants. Although pro-MMP-2 and MMP-2 bands were observed in the supernatants of normal myoblasts, only pro-MMP-2 was detected in DM1 myoblast supernatants. The combined levels of pro-MMP-2 and MMP-2 in DM1 myoblasts culture supernatants was 47% lower than that in normal myoblast supernatants (Figure 3a). We next assayed whether blocking KCa1.1 was sufficient to induce the reduction in MMP-2 levels observed in DM1 supernatants. Paxilline treatment induced an approximately 40% decrease in combined pro-MMP-2 and MMP-2 levels in normal myoblast supernatants to levels comparable to those observed in DM1 supernatants. In contrast, paxilline exerted no significant effect on pro-MMP-2 levels in DM1 myoblast supernatants (Figure 3b). The addition of CsA rescued the supernatant levels of MMP-2 inhibited by KCa1.1 block in normal myoblasts (Figure 3c). In DM1 myoblast supernatants, neither paxilline nor CsA significantly altered the pro-MMP-2 levels. However when CsA was added to the DM1 myoblasts with paxilline, MMP-2 levels in the secretion showed a trend towards efficacy with an increase by 67% although analysis showed no statistical effect (Figure 3d).

### KCa1.1 block alters myoblast cell cycle distribution

**Figure 4:**
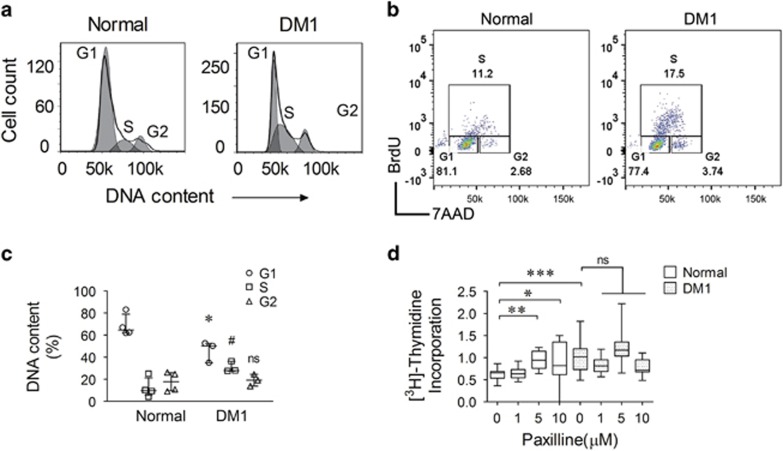
KCa1.1 channel block enhances the proliferation of myoblasts. (**a**) Representative flow cytometric cell cycle profiles (G1/S/G2) of normal (left) and DM1 (right) myoblasts fitted to the Dean-Jett model. (**b**) BrdU/7-AAD cell cycle analysis of normal and DM1 myoblasts. (**c**) Percentage of cells in each phase of the cell cycle for normal (*n*=4 independent replicates) and DM1 (*n*=3 independent replicates) myoblasts. *P<0.05 by Mann–Whitney *U*-test comparing G1-phase between normal and DM1 myoblasts expressed as; ^#^*P*<0.05 by Mann–Whitney *U*-test comparing S-phase between normal and DM1 myoblasts; NS, not significant. (**d**) [^3^H] thymidine incorporation assay showing the proliferation of normal (black; *n*=18 independent replicates, four donors) and DM1 (white; *n*=18 independent replicates, three donors) myoblasts treated with increasing concentrations of paxilline or vehicle (DMSO). Median and interquartile ranges values normalized to the proliferation of vehicle-treated DM1 myoblasts (6895±822 c.p.m.). *P<0.05; **P<0.01; ***P<0.001; NS, not significant by Kruskal–Wallis test with Dunn's multiple comparisons

During the early stages of myogenesis, the rate of cell division and the events leading to myoblast differentiation into myotubes are highly orchestrated by myogenic transcription factors and growth factors, including NF*κ*B.^27, 34, 35, 36, 37^ As KCa1.1 block induced an increase in levels of activated NF*κ*B in normal myoblasts, and NF*κ*B was shown to regulate progression through the cell cycle in myoblasts,^27^ we assessed the effects of the loss of KCa1.1 function on the cell cycle distribution of myoblasts. Approximately 60–80% of normal myoblasts were in the G1-phase of the cell cycle and fewer than 25% were in the S-phase (Figures 4a and c). In comparison, a higher proportion of DM1 myoblasts were in the S-phase of the cell cycle (25–37%) and fewer than 55% were in the G1-phase. To further assess the myoblasts' cell cycle status, 5-bromo-2′-deoxyuridine (BrdU) was added to directly stain the newly synthesized DNA. DM1 myoblasts showed increased BrdU staining compared with the normal myoblasts (Figure 4b) suggesting that more DM1 myoblasts than normal myoblasts are in the DNA replicative stage of the cell cycle.

### KCa1.1 block enhances myoblast proliferation

The inability of myoblasts to stop proliferating is known to delay myotube fusion.^7, 38^ To determine whether the differences in cell cycle between normal and DM1 myoblasts is reflected in different proliferation rates between these cells, we measured [^3^H]-thymidine incorporation into the DNA of dividing myoblasts.^39^ As expected,^7, 38^ DM1 myoblasts had a significantly higher proliferative rate than normal cells (Figure 4d). We next tested the effects of paxilline on myoblast proliferation. Although paxilline did not affect DM1 myoblast proliferation, it did enhance that of normal myoblasts to the level of untreated DM1 myoblasts (Figure 4d).

### KCa1.1 channel block inhibits the fusion of normal myoblasts into myotubes

**Figure 5:**
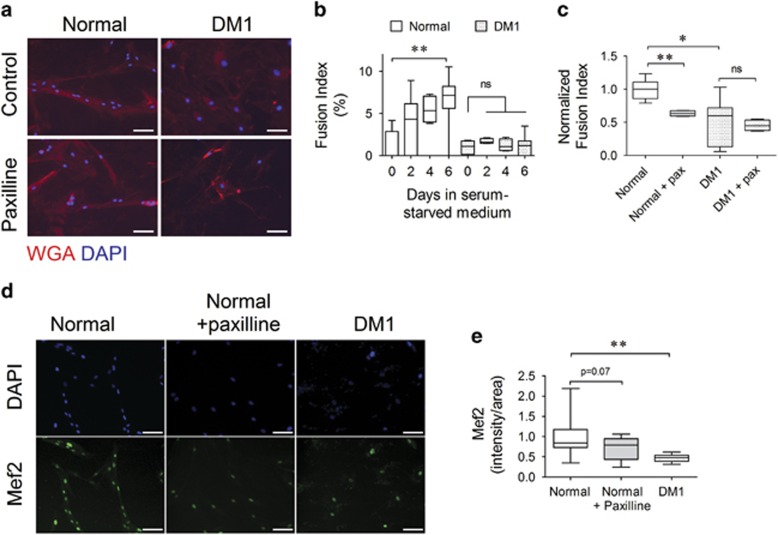
KCa1.1 channel block inhibits the fusion of normal myoblasts into myotubes. (**a**) Representative images of myotubes after 6 days in fusion medium containing the vehicle (control) or paxilline (pax; 10 *μ*M) and stained with WGA-Alexa 555 to visualize the plasma membrane (red) and DAPI to visualize nuclei (blue). Scale bar=100 *μ*m. (**b**) Fusion index (%) of normal (black, *n*=3 independent replicates) and DM1 (white, *n*=4 independent replicates) myoblasts after 0, 2, 4, and 6 days in fusion medium expressed as median and interquartile ranges. *P<0.05; NS, not significant by Kruskal–Wallis test with Dunn's multiple comparisons. (**c**) Fusion index of normal (black, *n*=4 independent replicates) and DM1 (white, *n*=3 independent replicates) myoblasts treated with vehicle or with paxilline (10 *μ*M) for 6 days and normalized to the fusion index of vehicle-treated normal myotubes expressed as median and interquartile ranges. *P<0.05; **P<0.01; NS, not significant by Kruskal–Wallis test with Dunn's multiple comparisons. (**d**) Representative images of myoblasts treated with paxilline, incubated in fusion medium for 6 days and stained with antibody against mef2 (green) and DAPI (blue). (**e**) The histogram shows nuclear image intensity of mef2 expressed as median and interquartile ranges (*n*=8 replicates, three donors; three to six images per slide). **P<0.01 by Kruskal–Wallis test with Dunn's multiple comparisons

During myogenesis, myoblasts fuse to form multinucleated myotubes, a process regulated by NF*κ*B.^27^ To test the effects of modulating KCa1.1 on their myogenic capacity, myoblasts were cultured in low-serum fusion medium with paxilline, and multi-nucleation of myotubes was analyzed. Normal myotubes showed multi-nucleation (>3 nuclei per myotube) at the end of the 6-day fusion assay, whereas DM1 cells remained mostly mononucleated (Figures 5a and b). The treatment of normal myoblasts with paxilline decreased myotube fusion by 36% (Figures 5a and c) and mef2 expression by 18% (Figures 5d and e), thus showing that the loss of functional KCa1.1 channels is sufficient to induce a decrease in myoblast fusion, as seen in DM1.

A tight regulation of membrane potential is necessary for myoblast fusion^40^ and potassium channels regulate the membrane potential of various cell types.^13, 41^ We therefore compared the membrane potential of normal and DM1 myoblasts by current clamp and detected no significant difference, with membrane potentials for both cell types around −60 mV (Supplementary Figure S3), as previously reported.^12^

**Figure 6:**
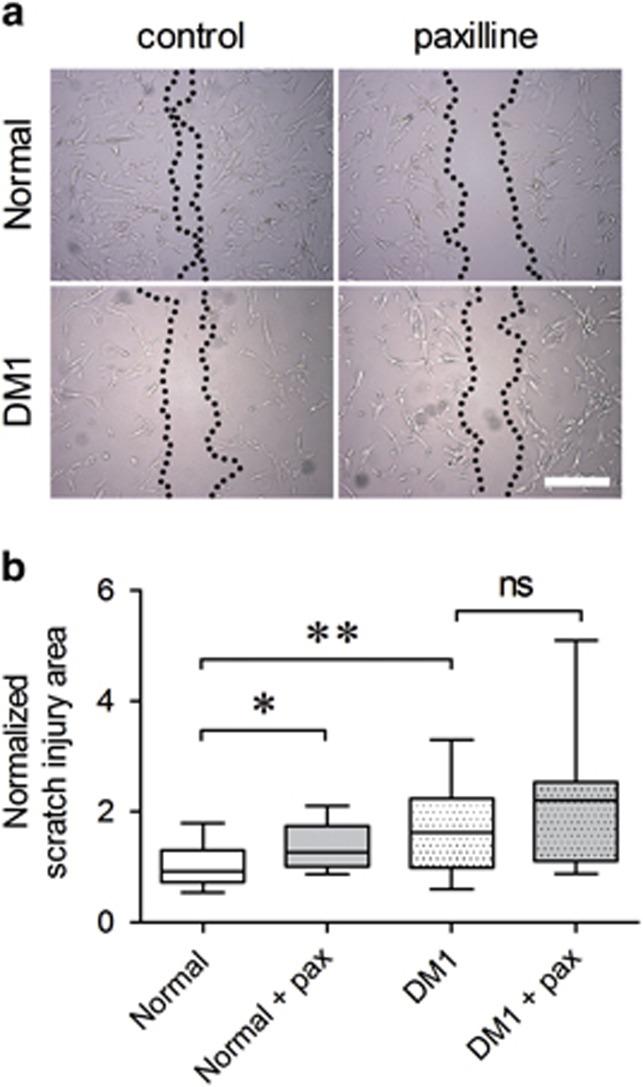
KCa1.1 channel block impairs myoblast migration. (**a**) Representative bright-field images of normal and DM1 myoblasts 6 h after drawing a scratch line and incubated in growth medium containing vehicle (DMSO control) or paxilline (10 *μ*M). The cell-free area is shown between two dotted black lines. Scale bar=400 *μ*m. (**b**) Median and interquartile ranges of the scratch injury area normalized to that obtained with vehicle-treated normal myoblasts. *N*=11 independent replicates, three donors. *P<0.05; **P<0.01; NS, not significant by Kruskal–Wallis test with Dunn's multiple comparisons

The fusion of myoblasts requires their migration towards one another. As modulating KCa1.1 function affected supernatant levels of MMP-2 and this MMP is necessary for myoblast migration, we assessed the effects of paxilline on myoblast migration in a wound-healing assay. Normal myoblasts were 40% more migratory than DM1 myoblasts. Blocking KCa1.1 with paxilline reduced normal myoblast migration to levels similar to those of DM1 myoblasts, whereas paxilline had no significant effect on DM1 myoblast migration (Figures 6a and b). Similar results were obtained by treating myoblasts with the nonselective potassium channel blocker TEA or with another selective KCa1.1 channel blocker, iberiotoxin (Supplementary Figures S4a and b).

### Introducing KCa1.1 channels into DM1 myoblasts rescues their altered proliferative rate and improves their fusion

**Figure 7:**
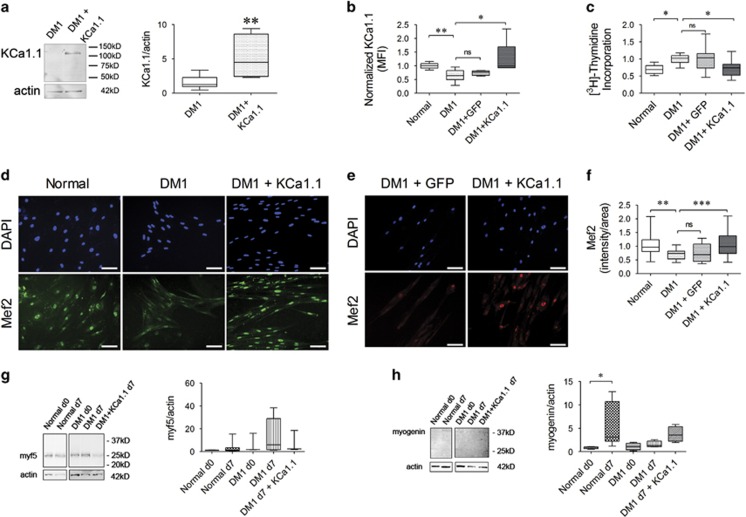
Introducing KCa1.1 channels into DM1 myoblasts rescues them from altered proliferation and improves fusion. (**a**) Representative western blot of KCa1.1 (green, IR800) and actin (red, IR680) in DM1 myoblasts with or without KCa1.1-expressing baculovirus transduction. The histogram shows median and interquartile ranges of protein level of KCa1.1 normalized to actin (*n*=5 replicates). **P<0.01 by Mann–Whitney *U*-test comparing normal with DM1. (**b**) Median fluorescence intensities (MFI) and interquartile ranges of KCa1.1 *α* staining in normal, DM1 and DM1 with GFP or KCa1.1-expressing BacMam baculovirus transduction (*n*=5 independent replicates) myoblasts compared with negative control; *P<0.05 by Kruskal–Wallis test with Dunn's multiple comparisons. (**c**) [^3^H]-thymidine incorporation assay of normal, DM1 and DM1 with GFP or KCa1.1-expressing BacMam baculovirus transduction (*n*=5 independent replicates, three donors). The histogram shows median and interquartile ranges values normalized to the proliferation of untreated DM1 myoblasts. *P<0.05 by Kruskal–Wallis test with Dunn's multiple comparisons. (**d**) Representative images of normal and DM1 myoblasts after KCa1.1 *α*-expressing BacMam transduction stained with myogenic enhancer factor, mef2 (green) and counterstained with DAPI (blue). Scale bar=100 *μ*m. (**e**) Representative images of DM1 myoblasts transduced with GFP-expressing BacMam and KCa1.1 *α*-expressing BacMam, and stained with mef2 (red) and DAPI (blue). (**f**) The histogram shows image intensity of Mef2 in nuclear region expressed as median and interquartile ranges (*n*=9 independent replicates, three donors). *P<0.05 by Kruskal–Wallis test with Dunn's multiple comparisons. (**g** and **h**) Representative western blot of myf5 (**g**) and myogenin (**h**) with actin in total protein extracts from normal myoblasts (d0), normal myotubes (d7), DM1 myoblasts (d0), DM1 myotubes (d7), and DM1+KCa1.1 myotubes (d7). The histograms shows median and interquartile ranges of myf5 and myogenin protein levels normalized to actin. *N*=4 replicates; *P<0.05 by Kruskal–Wallis test with Dunn's multiple comparisons

As DM1 myoblasts express fewer functional KCa1.1 channels at their plasma membrane than normal myoblasts and blocking KCa1.1 in normal myoblasts enhanced their proliferation and impaired fusion, we sought to determine whether introducing KCa1.1 channels into DM1 myoblasts would rescue myogenesis. We transduced DM1 myoblasts with a KCa1.1 *α*-expressing BacMam baculovirus^42^ and confirmed enhanced expression of KCa1.1 channels by western blot (Figure 7a) and FCM (Figure 7b).

Transduction of DM1 skeletal myoblasts with a KCa1.1*α*-expressing BacMam baculovirus, but not a GFP-expressing BacMam baculovirus, significantly decreased their proliferation to levels comparable to those of normal myoblasts (Figure 7c). This decrease in proliferation of KCa1.1 *α*-transduced DM1 myoblasts suggests that these myoblasts are now likely fusion-competent.^7, 43^ We therefore tested their ability to fuse into multinucleated myotubes. Normal myoblasts differentiated into myotubes with nuclear expression of the late myogenic marker mef2. In contrast, the expression of mef2 was lower in DM1 myotubes (Figures 7d and f). DM1 myoblasts transduced with KCa1.1 *α*, but not those transduced with GFP, significantly increased their mef2 expression (Figures 7e and f) thus demonstrating that the introduction of KCa1.1 is sufficient to induce fusion of DM1 myoblasts into myotubes when cultured in fusion medium. We also checked earlier events in myogenesis. The protein levels of myf5 did not change significantly during myotube fusion in either normal or DM1 myoblasts. However, we observed higher myf5 levels in DM1 myoblasts/myotubes suggesting the presence of highly proliferative DM1 cells even at day 7 of fusion assays (Figure 7g). DM1 myotubes that received KCa1.1-BacMam showed a 42% decrease in myf5 compared with untreated ones (Figure 7g). DM1 myotubes transduced with KCa1.1-BacMam showed a 3.5-fold increase in myogenin levels compared with untreated DM1 myotubes, the latter displaying only 1.5-fold increase in myogenin expression from day 0 myoblasts (Figure 7h). Therefore, transduction of KCa1.1*α* into DM1 myoblasts during fusion assays induces a modest decrease in myf5 and increase in myogenin.

## Discussion

We demonstrated the loss of functional KCa1.1 at the plasma membrane of myoblasts obtained from patients with DM1. We showed that KCa1.1 regulates calcium homeostasis and NF*κ*B activation; KCa1.1 is crucial in maintaining appropriate proliferation, migration, and myotube fusion of normal myoblasts. Our work suggests a novel approach for targeting muscle wasting in DM1 by identifying myogenic events disturbed by the loss of KCa1.1.

Our data demonstrate a decreased expression of KCa1.1 at the plasma membrane of DM1 skeletal myoblasts. However, we and others^44^ showed no significant difference in the resting membrane potential between normal and DM1 myoblasts. The loss of functional KCa1.1 may be compensated by the upregulation of other ion channels in DM1 myoblasts, sufficient to maintain the normal membrane potential but insufficient to replace KCa1.1 in regulating myoblast proliferation and fusion. Indeed, although paxilline inhibited the majority of potassium currents at the plasma membrane of normal myoblasts, it only reduced those of DM1 myoblasts by less than 25%. These results suggest that, although KCa1.1 is the major potassium channel at the plasma membrane of normal myoblasts, DM1 myoblasts express a higher ratio of paxilline-resistant ion channels over KCa1.1. The future identification of these channels may provide information on their compensatory upregulation, if any, and why their expression is unable to compensate for the loss of KCa1.1 in regulating myoblast proliferation and differentiation.

Western blots using whole-cell lysates and immunocytochemistry using fixed cells permeabilized with Triton X-100 detect all KCa1.1 *α*-subunits, regardless of the subcellular localization. In contrast, patch-clamp assays focused on channels expressed at the plasma membrane of the myoblasts. FCM involved saponin-based cell permeabilization before the detection of KCa1.1 *α*. This detergent removes membrane cholesterol; it is therefore not effective in permeabilizing cholesterol-poor membranes, such as the nuclear membrane.^45^ The lower levels of KCa1.1 *α* detected by FCM when compared with immunocytochemistry is therefore likely owing to the localization of a large pool of KCa1.1 *α* in the nucleus of the myoblasts. A nuclear localization of this channel has been described in other cells^46^ and the roles of nuclear KCa1.1 in myoblasts will have to be dissected.

Although a decrease in KCa1.1 *α*-subunit was observed at the functional and protein expression levels, no such differences were observed at the level of mRNA expression when using primers directed to a conserved region of the *α*-subunit of KCa1.1. DM1 is considered an RNA-mediated disease, with the pathogenesis of this disease in large part from dysregulation of alternative splicing programs.^47, 48, 49^ KCa1.1 *α* contains multiple alternating splicing sites with more than 30 splice variants identified in different tissues and species.^16, 50, 51^ Alternative splicing of KCa1.1 *α* can affect not only the potassium-conducting properties of the channel but also its interactions with signaling molecules and its subcellular localization. Immunocytochemistry on skeletal myoblasts showed a loss of plasma membrane expression of KCa1.1 *α* in DM1 cells, but these cells retained its nuclear expression. Transduction of DM1 myoblasts with full-length KCa1.1 *α* increased not only total protein levels but also plasma membrane expression of the channel. Taken together, these data suggest that the mechanism of reduced plasma membrane expression of KCa1.1 in DM1 myoblasts is not a result of defective protein trafficking but rather of alternative splicing or other modifications of KCa1.1, leading to its aberrant subcellular localization. Alternatively, the difference in KCa1.1 localization in normal and DM1 myoblasts could be a result of altered expression or splicing of regulatory *β*- or *γ*-subunits. Indeed, these subunits affect not only the activation kinetics of the channel and its response to intracellular calcium levels, but also the subcellular localization of KCa1.1 *α*.^16, 50, 52^ Although the current study focuses on the consequences of the loss of plasma membrane expression of KCa1.1 in DM1 myoblasts, future work aimed at identifying the splice variants of *α-*, *β*-, and *γ*-subunits of KCa1.1 in normal and DM1 myoblasts is needed to understand the causes of this loss. This would not be the first finding of alternative splicing of an ion channel in DM1 myoblasts. Indeed, the sustained influx of calcium that was observed in DM1 myoblasts has been attributed to the misregulated alternative splicing and altered gating of the calcium channel Cav1.1.^10^ Other reports have attributed increased intracellular calcium in resting DM1 myotubes from patients to a decrease in RyR1 and increase in calcium influx through Cav1.1.^12, 53, 54^ Although calcium channels directly mediate calcium influx, potassium channels are key regulators of membrane potential and cell volume; their function therefore also affects intracellular calcium concentrations.^55^ In turn, intracellular calcium regulates transcription factors necessary for myotube fusion in both C2C12 and human myoblasts.^40^

The varied sources of intracellular calcium could affect different cell activities. The two major sources of intracellular calcium in myoblasts are influx via ion channels and release from internal stores. Steady increase in intracellular calcium activates the calcium–calcineurin pathway, induces NF*κ*B activation, and regulates mef2.^56^ In contrast, short spikes of calcium transients from the internal stores regulate localization and function of myogenin and mef2 without affecting myf5.^57^ Interestingly, altering KCa1.1 function or expression induced a change in mef2 and myogenin levels with no significant changes in myf5 levels. Although our results point to a functional link between KCa1.1 expression and function and calcium homeostasis in human myoblasts, KCa1.1 is likely not the only regulator of basal calcium levels in these cells as multiple pathways converge onto calcium as a signaling molecule. Future studies are required to identify the source of cytosolic calcium affected by KCa1.1 function in myoblasts, identify the calcium-permeable channels responsible for calcium influx, and fully dissect the signaling pathways that regulate calcium homeostasis in these cells.

Paxilline-sensitive potassium channels were present at the plasma membrane of both normal and DM1 myoblasts, albeit at low levels in the latter. However, paxilline affected the proliferation, MMP-2 culture supernatant levels, and fusion of normal myoblasts but not of DM1 myoblasts. This suggests that the KCa1.1 remaining at the plasma membrane of DM1 myoblasts are insufficient to regulate the signaling pathways that control cell proliferation and fusion.

Blocking KCa1.1 in normal myoblasts induced a decrease in pro-MMP-2 and MMP-2 levels to those obtained with DM1 myoblasts. These results are in agreement with studies showing that functional plasma membrane potassium channels are necessary for protease production in different tissues.^19, 42^ CsA rescued the decrease in MMP-2 levels in supernatants from paxilline-treated normal myoblasts showing that the KCa1.1-dependent regulation of MMP-2 production or secretion is regulated by calcium–calcineurin dependent mechanisms. However, in DM1 myoblasts, CsA alone did not rescue MMP-2 levels. Interestingly, when CsA was applied in combination with paxilline, a 67% increase in MMP-2 levels was observed. Taken together, these results show that the regulation of pro-MMP-2 and MMP-2 levels released in myoblast supernatants is regulated in part by KCa1.1 and calcium–calcineurin and also by calcineurin-independent mechanisms.

Blocking KCa1.1 in normal myoblasts induced an increase in proliferation, similar to that observed with DM1 myoblasts, whereas overexpression of full-length KCa1.1 *α* in DM1 myoblasts reduced their proliferation to levels observed in normal myoblasts. Interestingly, paxilline also induces the proliferation of some cancer cells expressing KCa1.1.^58^ However, in other tissues, blocking KCa1.1 and other potassium channels inhibits proliferation.^13, 19, 42, 50^ This discrepancy between tissues underlines a differential role of these channels dependent on the different signaling pathways that lead to cell proliferation.

Blocking KCa1.1 recapitulated some of the DM1 phenotype in normal myoblasts and, in reverse, the transduction of DM1 myoblasts with full-length KCa1.1 *α* restored normal proliferation and fusion of DM1 myoblasts. Taken together, our data identify KCa1.1 as crucial regulators of myoblast proliferation and fusion and suggest these channels as potential targets for correcting delayed myogenesis in DM1.

## Materials and Methods

### Human myoblasts

Primary skeletal myoblasts from healthy volunteers and DM1 patients were obtained from muscle biopsy samples with Institutional Review Board approval. All the samples were tested for myoblast purity using myogenic markers. Primary myoblasts from control patients with normal muscle histology and normal metabolism and from DM1 patients with more than 300 CTG repeats were used. The cells were cultured at 37 ºC, 5% CO_2_, in high-serum growth medium containing F-10/Ham nutrient (Sigma, St Louis, MO, USA), 15% fetal bovine serum, 5% bovine supplemental calf serum, and 1% penicillin/streptomycin/glutamine/amphotericin.^59^

### KCa1.1 channel modulation

The small molecule paxilline (Tocris, Ellisville, MI, USA) and the peptide iberiotoxin (CS Bio, Menlo Park, CA, USA) were used as a selective blocker of KCa1.1.^60^ Tetraethylammonium (TEA) chloride (Sigma) was used as a nonselective blocker of potassium channels. The *α*-subunits of KCa1.1 were transduced into myoblasts using the Bacmam expression system as described;^42, 61^ BacMam vectors expressing GFP were used as controls. Myoblasts were transduced at a multiplicity of infection of 100.

### Western blot

Total proteins were extracted in RIPA lysis and extraction buffer supplemented with 1% protease inhibitor cocktail (Roche, Indianapolis, IN, USA). The protein levels were measured using the Bradford protein assay and 15 *μ*g of proteins were loaded onto an SDS-PAGE gel and separated before transfer onto a nitrocellulose membrane. The blots were probed using mouse monoclonal anti-KCa1.1 (clone L6/60; UC Davis/NIH Neuromab Facility) or rabbit polyclonal anti-NF*κ*B p65 (Santa Cruz Biotechnology, Dallas, TX, USA) antibodies and rabbit monoclonal antibody against *β*-actin (Sigma). The blots were further incubated with secondary antibodies conjugated to fluorophores (LI-COR, Lincoln, Nebraska) and visualized using an Odyssey Imaging System (LI-COR).

### KCa1.1 *α* expression and cell cycle analyses by FCM

The myoblasts were harvested and fixed in PBS+1% PFA. They were then either (i) 0.5% saponin-permeabilized and stained with mouse monoclonal anti-KCa1.1 *α* antibodies (NeuroMab, Davis, CA, USA) to detect channel levels as described^42^ or (ii) 0.1% Triton X-100 permeabilized and stained with 7-amino actinomycin D (7-AAD) to label DNA for cell cycle analysis. In addition, 5-bromo-2′-deoxyuridine (BrdU) was used to label cells in culture before 7-AAD staining following the manufacturer's protocol. Fluorophore-conjugated secondary antibodies were used where appropriate (Invitrogen, Grand Island, NY, USA). The data were acquired with a Becton Dickinson Canto II flow cytometer and analyzed with FlowJo Version 7 (Treestar, Ashland, OR, USA). The Dean-Jett model was applied to evaluate G1/S/G2-phase DNA content (%).^62^ Analysis of cell cycle was performed with asynchronous population of cells because serum-starvation led to the induction of cell fusion into multinucleated cells, thereby directly affecting their cell cycle.

### Immunocytochemistry

The myoblasts were fixed with phosphate-buffered saline (PBS)+1% paraformaldehyde (PFA), permeabilized with freshly prepared PBS+5% bovine serum albumin+2% goat serum+0.1% Triton X-100, then incubated with primary antibodies: mouse monoclonal anti-KCa1.1 (clone L6/60; UC Davis/NIH Neuromab Facility) or rabbit polyclonal anti-Mef2 (Santa Cruz Biotechnology) or rabbit polyclonal anti-NF*κ*B p65 (Santa Cruz Biotechnology), all at a final concentration of 10 *μ*g/ml. They were next incubated with appropriate fluorophore-conjugated secondary antibodies (Alexa Fluor 488 goat anti-mouse IgG or Alexa Fluor 488 goat anti-rabbit IgG; Invitrogen). The slides were mounted with SlowFade Gold anti-fade mountant with DAPI (Invitrogen). Staining was detected with a Zeiss LSM 510 inverted laser scanning microscope (Carl Zeiss, Thornwood, NJ, USA) at room temperature with × 10 and × 40 objective lenses.

### Electrophysiology

The myoblasts were plated at a low density to avoid cell–cell contacts onto sterile glass coverslips and allowed to adhere before whole-cell path-clamp. The internal solution contained 145 mM KF, 10 mM HEPES, 10 mM EGTA, and 2 mM MgCl_2_. The pH of the internal solution was adjusted to 7.4 with KOH. The external (bath) solution contained 160 mM NaCl, 4.5 mM KCl, 2 mM CaCl_2_, 1 mM MgCl_2_, and 10 mM HEPES.^63^ The pH of the bath solution was adjusted to 7.4 with NaOH. Osmolarity of all solutions was adjusted to 290–320 mOsm. Whole-cell currents were measured at room temperature (20–25°C) with a holding potential of −80 mV. For testing ion channel modulators, the whole-cell patch was pulsed with 200 mV test potentials for 50 ms every 10 s.

### Semi-quantitative RT-PCR

Total RNA was isolated from myoblasts using TRIzol (Invitrogen) and cDNA was synthesized using oligo dT and Superscript II reverse transcriptase. The resulting cDNA was used as a template for PCR with primers for a conserved region of the KCa1.1 *α* subunit. GAPDH was used as a control. The primer sequences^19^ used were: KCa1.1 *α* Forward: 5′-ACAACATCTCCCCCAACC-3′ and Reverse: 5′-TCATCACCTTCTTTCCAATTC-3′ GAPDH Forward: 5′-CGATGCTGGGCGTGAGTAC-3′ and Reverse: 5′-CGTTCAGCTCAGGGATGACC-3′. The PCR products were separated in a 1% agarose gel and visualized under UV light. Quantification was done by analysis of the scanned gels with ImageJ.

### mRNA stability assay

Normal and DM1 skeletal myoblasts were cultured in myoblasts growth medium in the presence or absence of 20 *μ*g/ml of actinomycin D. At 0, 2, 4, 6, and 8 h, the cells were lysed in Trizol reagent (Thermo Fisher Scientific, Rochester, NY, USA), and total RNA was isolated as per the manufacturer's instructions. Total RNA was transcribed to cDNA using the Superscript III First-Strand Synthesis System (Thermo Fisher Scientific) as per the manufacturer's instructions, priming with random hexamers. The cDNA obtained from this reaction was subjected to quantitative PCR using the Kapa Sybr Fast System (Kapa Biosystems, Willmington, MA, USA) and the following oligonucleotide primers: KCNMA1_Forward: 5′-CCTCCTCCATGGTGACTTTC-3′ and_Reverse: 5′-GCACACGGTCCACAGGTACT-3′ TATA-box binding protein (TBP) Forward: 5′-TCAAACCCAGAATTGTTCTCC-3′ and Reverse: 5′-GAGCCATTACGTCGTCTTCC-3′ (Sigma-Aldrich, St Louis, MO, USA). Relative expression of KCNMA1 mRNA over time was normalized to TBP expression and analyzed using standard DDCt methodologies.

### Cytotoxicity

The myoblasts were incubated with paxilline (1, 5, 10, and 100 *μ*M) or staurosporine (0.1 and 1 *μ*M; positive control) for 16 h in high-serum growth medium. The cells were then collected, washed, and stained with 7-amino actinomycin D (7-AAD) to label DNA in non-viable cells as described.^19, 64^ The data were acquired with a Canto II flow cytometer and analyzed using FlowJo Version 7.

### [^3^H]-Thymidine incorporation assays

The myoblasts were plated into high-serum growth medium in 96-well plates (10 000 cells per well). Paxilline or vehicle was immediately added at the indicated concentration and the cells were cultured for 72 h at 37 ºC, 5% CO_2_. [^3^H] thymidine was added during the last 16–18 h to label replicating DNAs of dividing cells. The cells were lysed by freezing at −20 °C followed by the addition of distilled H_2_O to the wells. DNA of lysed cells was harvested onto glass fiber filters. The amount of [^3^H] thymidine incorporation into the DNA of dividing cells was counted using a *β*-scintillation counter, as described,^19, 63, 65^ as a measure of cell proliferation.^39^

### Gelatin-gel zymography

The myoblasts were plated in six-well plates in high-serum growth medium until adherent. The medium was removed and the cells were washed with PBS before the addition of 0.5 ml DMEM supplemented with 2% fetal bovine serum. The myoblasts were then incubated in the new medium, with paxilline, cyclosporin A, or vehicle for 24 h and the supernatants were collected for running gelatin-gel zymographies, as previously described.^66^

### Intracellular calcium measurement

The measurements were conducted both by FCM and fluorescence microscopy. For FCM, the myoblasts were incubated with the ratiometric calcium indicator dye Indo-1-AM (2 *μ*M; Invitrogen) for 30 min at 37 ºC, washed twice with PBS containing calcium and magnesium, and resuspended in 0.5 ml PBS for 30 min to allow for de-esterification of the AM. The basal intracellular calcium signal was measured using a Becton Dickinson LSRII flow cytometer by exciting Indo-1 with a UV laser (350 nm) and measuring the ratio of emissions at 379/28 nm (calcium-bound) and at 480/40 (calcium-free) at room temperature. After a stable baseline was acquired, the Indo-1 signal was continuously recorded for 5 min after the addition of 10 *μ*M paxilline. At the end of each assay, 1 *μ*M ionomycin was added to assess the maximal intracellular calcium level in the myoblasts used.^18^

For fluorescence microscopy, the myoblasts were plated onto glass coverslips and incubated with Indo-1-AM (2 *μ*M) for 30 min at 37 ºC, washed with PBS containing calcium and magnesium, and incubated in PBS for 30 min to allow de-esterification of the AM. The coverslip was placed on the stage of an AE31 inverted microscope (Motic, Richmond, BC, Canada) equipped with a × 40 Olympus UApo 340 objective. Indo-1 excitation (340 nm) and emission (395/480 nm) were monitored using the IonOptix Myocyte Calcium and Contractility Recording System (IonOptix, Westwood, MA, USA). The acquisition of Indo-1 ratiometric data was done at room temperature at the sampling rate of 250 Hz.

### Wound healing

The myoblasts were plated in four-chamber slides (Thermo Fisher Scientific) to 80% confluency in growth medium. A scratch line was drawn through the cell monolayer using a 200-*μ*l pipette tip. Lifted myoblasts were washed away with growth medium and the medium was replaced with growth medium with paxilline or vehicle.^19^ The myoblasts were cultured at 37 °C, 5% CO_2_, for 6 h and the scratch injury area was captured at room temperature using BX41 microscope (Olympus, Melville, NY, USA) equipped with Plan N × 10/0.25 objective lens and Qcapture software (Surrey, BC, Canada) linked to a Q-Color5 digital camera (Olympus). The area of scratch injury was measured using Adobe Photoshop CS3 (San Jose, CA, USA).

### Myoblast fusion

The cells were cultured to 80% confluency in high-serum growth medium and then switched to low-serum fusion medium containing DMEM supplemented with 0.01 M bovine insulin and 2% horse serum to induce differentiation.^59^ Fusion medium was replaced every other day for 6 days. At the indicated time points, the cells were stained with DAPI to detect nuclei, with wheat germ agglutinin to detect membrane structures, or with an antibody against mef2. The myotubes were identified as elongated structures containing three or more nuclei. For each condition, photos of five fields were taken. In each field of view, we counted the number of nuclei per myotube (*N*_myotube_) and the total number of nuclei (*N*_total_). The fusion index was calculated as (*N*_myotube_/*N*_total_) × 100.

### Statistical analysis

The results are expressed as median and interquartile ranges unless indicated otherwise in the figure legend. All statistical analyses were performed using GraphPad Prism (La Jolla, CA, USA). The tests used for the different assays are provided in the figure legends.

## Abbreviations

7-AAD: 7-amino actinomycin D
BK: big potassium channel
BrdU: 5-bromo-2′-deoxyuridine
CaV: voltage-gated calcium channel
CsA: cyclosporin A
CUG: cytosine uracil guanine
DAPI: 4′,6-diamidino-2-phenylindole
DM1: myotonic dystrophy type 1
DMEM: Dulbecco's modified eagle medium
FCM: flow cytometry
GFP: green fluorescent protein
IBTX: iberiotoxin
MEF2: myocyte enhancer factor-2
MFI: median fluorescence intensity
MMP: matrix metalloproteinase
Myf5: myogenic factor-5
MyoD: myogenic determinant
NF*κ*B: nuclear factor kappa B
Pax: paxilline
PFA: paraformaldehyde
RT-PCR: reverse transcription polymerase chain reaction
TBP: TATA-box binding protein
TEA: tetraethylammonium
WGA: wheat germ agglutinin
