# Supplementary material for: Functional KCa1.1 channels are crucial for regulating the proliferation, migration and differentiation of human primary skeletal myoblasts
Source: Cell Death Dis. 2016 Oct 20;7(10):e2426–. doi: 10.1038/cddis.2016.324 (PMC5133989; doi:10.1038/cddis.2016.324)
Supplement: Supplementary Figures [file cddis2016324x1.docx]

**Supplementary material:**

Tajhya et al.

**
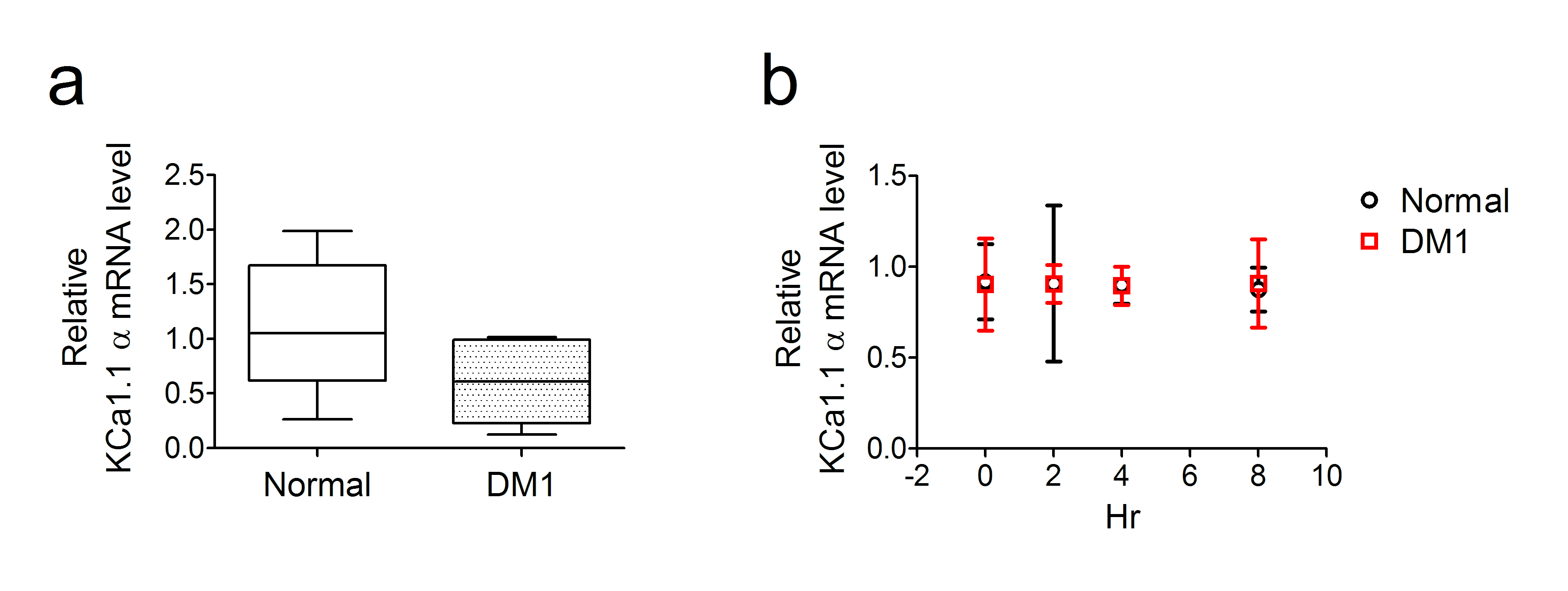
**

**Figure S1. The mRNA levels of KCa1.1 α in DM1 myoblasts are similar to that of normal myoblasts and are stable.** (**a**) KCa1.1 α mRNA expression normalized to GAPDH in normal (n=4) and DM1 (n=3) myoblasts by RT-PCR. P=0.0625 by Mann-Whitney *U* test. (**b**) The mRNA levels of KCa1.1 α in myoblasts treated with actinomycin D (10µg/mL) for 2, 4 and 8 hr were normalized to TBP and measured by quantitative PCR

**
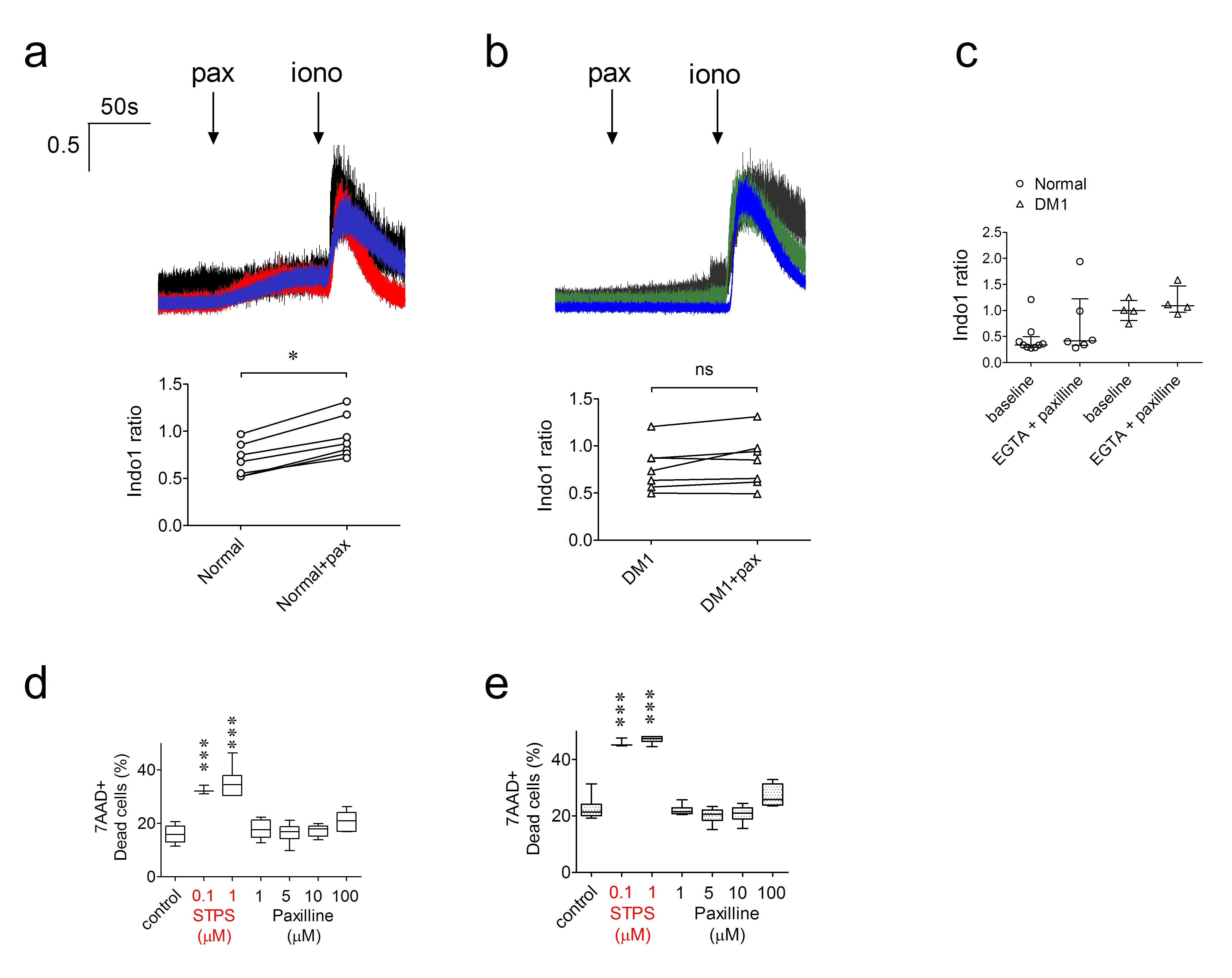
**

**Figure S2. Confirmation of paxilline effects on calcium influx by microscopy and lack of toxicity.** (**a** and **b**) Representative traces of ratiometric indo-1 measurements in normal (a) and DM1 (b) myoblasts by microscopy. Cells were treated with paxilline (10 µM) and ionomycin (1 µM) at the indicated times. Each cell’s response to paxilline is plotted in the before-after plot. N=4 normal and 2 DM1 donors; n=7 individual replicates. *p<0.05; ns= not significant by Wilcoxon matched-pairs test. (**c**) Myoblasts were pre-incubated with 5 mM EGTA for at least 1 hr before indo1 assay was performed. The changes in indo1 ratio are plotted as median and interquartile ranges. ns= not significant by Mann-Whitney test comparing baseline and EGTA+paxilline in normal (n=4 donors) and DM1 (n=3 donors) myoblasts. (**d** and **e**) Myoblasts were incubated with an increasing concentrations of staurosporine (0, 0.1, 1 µM STPS) and paxilline (1, 5, 10, 100 µM) over 48 hr. Staurosporine induced cytotoxicity at 0.1 and 1 µM; paxilline did not induce cytotoxicity even at the highest dose tested in normal (c) and DM1 (d) myoblasts. N=3 replicates; ***p<0.001 by Kruskal-Wallis test with Dunn’s multiple comparisons.

**
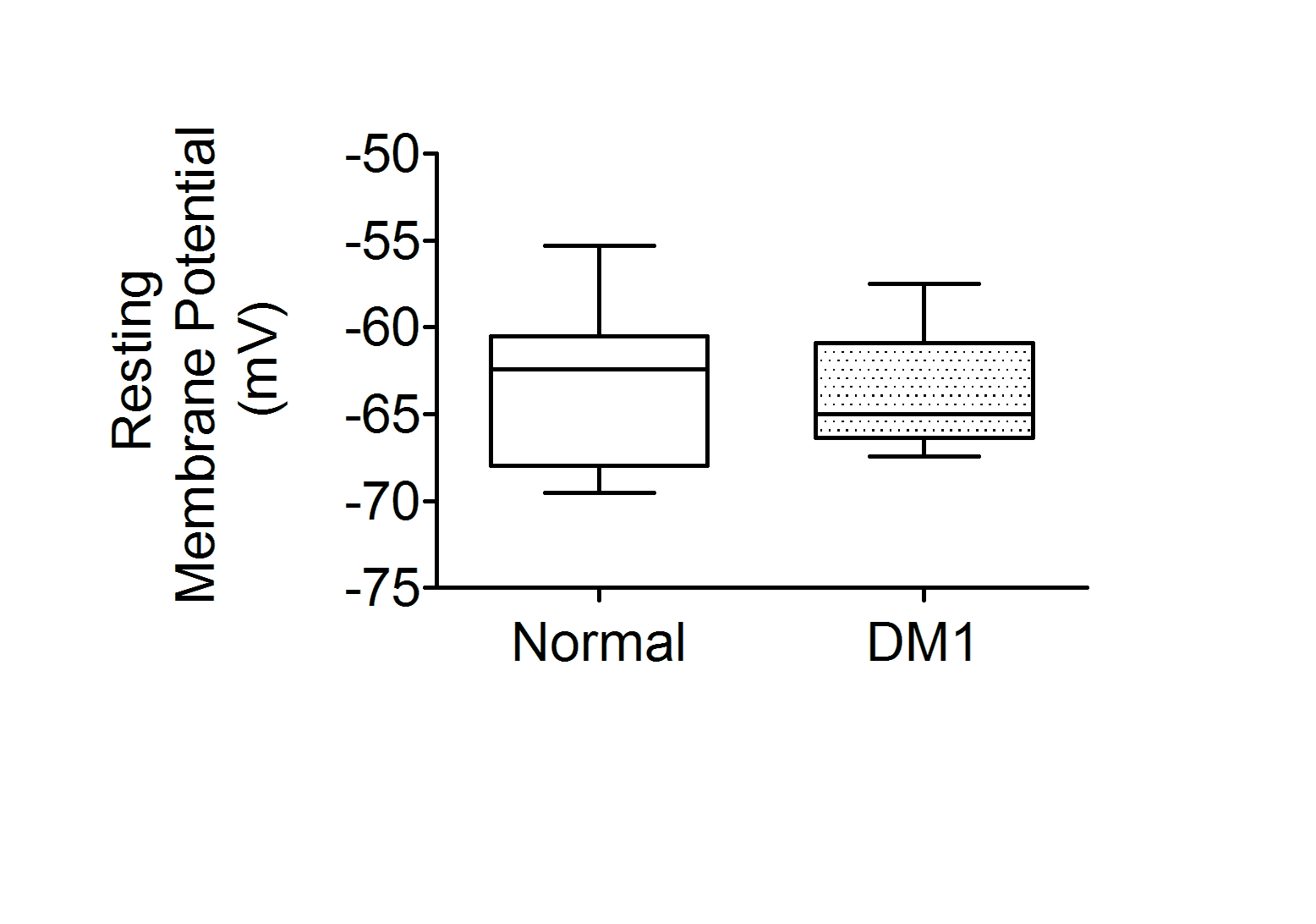
**

**Figure S3. The resting membrane potential on normal and DM1 myoblasts are similar.** Whole-cell current clamp recordings of normal (n = 12 cells) and DM1 (n = 5 cells) myoblasts showed a resting membrane potential of -62.42 (-67.96, -60.50) mV and -64.96 (-66.34, -60.88) mV, respectively. Data represented as median (25% percentile, 75% percentile).

**
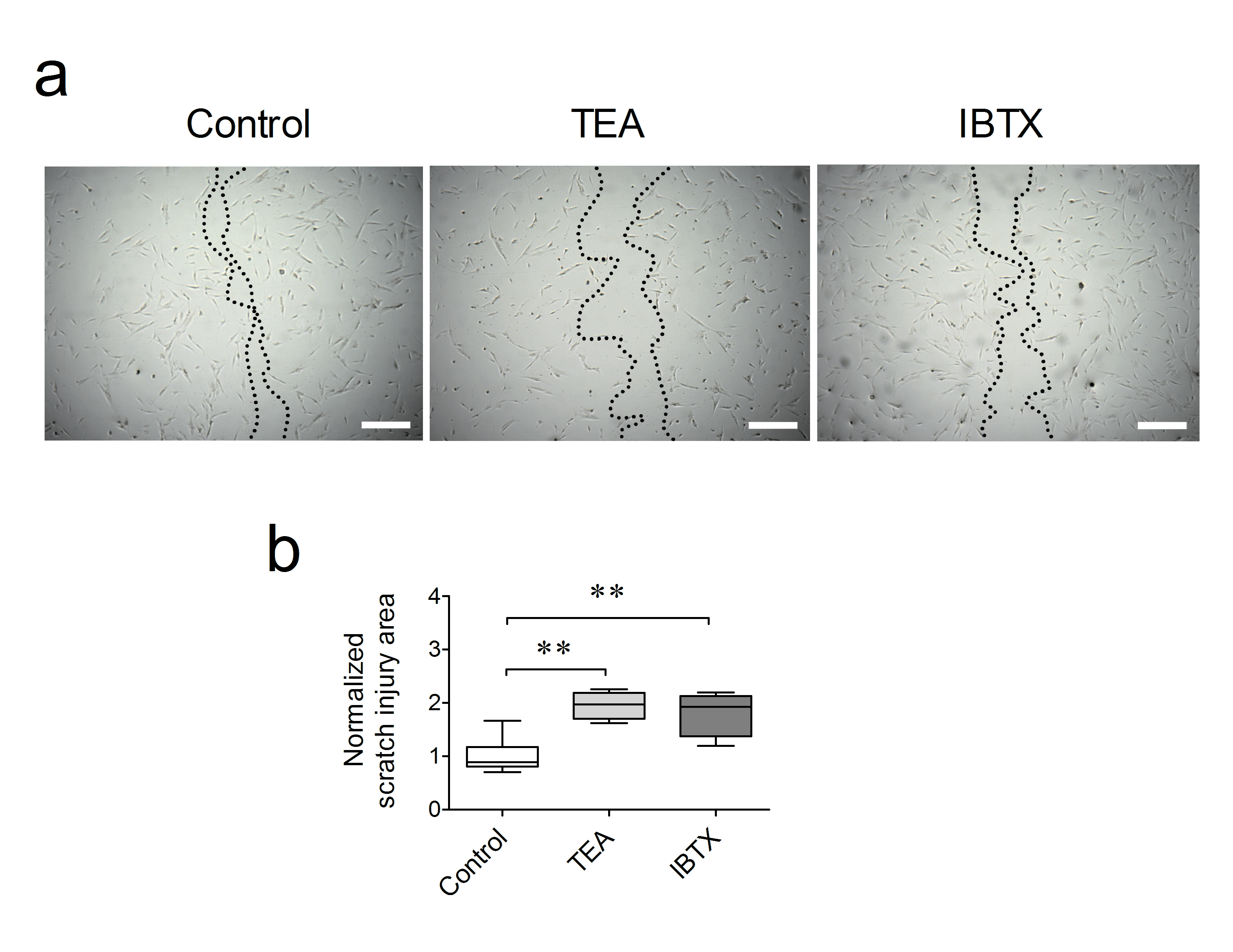
**

**Figure S4. K^+^ channel blockers, TEA and IBTX, delay scratch injury repair.** (**a**) Representative bright-field images of normal myoblasts 6 hours after drawing a scratch line and incubated in growth medium containing DMSO (control), tetraethylammonium (TEA, 1 mM) or iberiotoxin (IBTX, 10 µM). The scratch injury area is shown between the two dotted black lines. Scale bar = 400 μm. (**b**) Median and interquartile ranges of the scratch injury area normalized to that of control myoblasts. N=4 replicates; **p<0.01; ns = not significant by Kruskal-Wallis test with Dunn’s multiple comparisons.

**Supplementary video 1 and video 2. Immunostaining of KCa1.1 channels in normal and DM1 myoblasts.** The videos show a 3D rendering of Z-stacks (5.21 µm thick with 0.20 µm step size in Z) visualizing KCa1.1 channels (green) throughout the cytoplasm and nucleus in normal myoblast (Video 1) and mostly in nucleus in DM1 myoblast (Video 2). Nuclei are stained with DAPI (blue). Scale bar = 50 µm.
